# Imaging-based fibrosis assessment and risk stratification in MASLD

**DOI:** 10.3389/fmed.2026.1880238

**Published:** 2026-07-06

**Authors:** Shunqin Jin, Tao Zhou, Dachuan Jin, Jiahao Xie, Guoping Sheng, Hongzhi Li, Guangming Li, Chuan Qin, Peng Gao

**Affiliations:** 1 Department of Nuclear Medicine, The First Medical Center, Chinese PLA General Hospital, Beijing, China; 2 Department of Geriatric Medicine & Laboratory of Gerontology and Anti-Aging Research, Qilu Hospital of Shandong University, Jinan, China; 3 Translational Medicine Research Center, Department of Tuberculosis, Hepatology, and Pharmacy, Zhengzhou Sixth People's Hospital, Zhengzhou, China; 4 Key Laboratory of Artificial Organ and Computational Medicine of Zhejiang Province, Shulan (Hangzhou) International Medical College, Zhejiang Shuren University, Hangzhou, China; 5 Department of Infectious Diseases, Key Laboratory of Artificial Organs and Computational Medicine of Zhejiang Province, Shulan (Hangzhou) Hospital, Shulan International Medical College, Zhejiang Shuren University, Hangzhou, China

**Keywords:** elastography, fibrosis, magnetic resonance elastography, MASLD, non-invasive tests, risk stratification

## Abstract

**Introduction:**

Metabolic dysfunction-associated steatotic liver disease (MASLD) is now one of the leading causes of chronic liver disease worldwide. Fibrosis stage is the principal determinant of liver-related outcomes and overall mortality, yet liver biopsy is unsuitable for broad use because of invasiveness, sampling variability, and limited value for longitudinal assessment.

**Areas covered:**

This review discusses imaging-based assessment of fibrosis in MASLD with emphasis on ultrasound elastography, magnetic resonance elastography, multiparametric MRI, and selected emerging tools. The discussion is organized around diagnostic performance, technical limitations, prognostic value, treatment monitoring, and integration with serum-based non-invasive tests. In addition, this review proposes a practical framework linking pathophysiological mechanisms, imaging biomarkers, and stepwise clinical decision-making pathways for risk stratification in MASLD.

**Expert opinion:**

Imaging in MASLD now serves more than a diagnostic function. Ultrasound elastography remains the practical first-line imaging test because it can be deployed at scale and incorporated into referral algorithms. Magnetic resonance elastography offers the highest diagnostic confidence and is most useful when first-line tests are indeterminate, technically unreliable, or discordant with the clinical picture. Recent clinical pathways increasingly combine serum markers, elastography, quantitative magnetic resonance imaging (MRI), and longitudinal risk assessment rather than relying on a single modality or threshold.

## 1 Introduction

Metabolic dysfunction-associated steatotic liver disease (MASLD) has become the most prevalent chronic liver disease worldwide, paralleling the rise in obesity, type 2 diabetes, and metabolic syndrome (1, 2). Fibrosis stage is the principal determinant of liver-related events and overall mortality, which makes accurate fibrosis assessment central to surveillance, referral, and treatment planning (3). Accurate fibrosis staging therefore matters at every level of care. It determines which patients can remain under conservative follow-up, which require specialist referral, which are candidates for pharmacologic therapy or clinical trials, and which need monitoring for complications (4, 5). Liver biopsy remains the reference standard, but its use is constrained by invasiveness, sampling variability, interobserver variation, and poor suitability for serial assessment (1, 6). These limitations have driven the shift toward non-invasive tests. Serum-based scores are useful for initial risk exclusion, particularly in large populations, but they do not characterize the liver directly and leave many patients in an indeterminate zone (6, 7). Imaging addresses this gap by quantifying stiffness, steatosis, and, in selected settings, fibroinflammatory activity (6, 8).

Clinical use of imaging in MASLD has moved beyond simple detection of advanced fibrosis. Quantitative imaging biomarkers are increasingly used to refine risk stratification, identify patients at higher risk of progression, and monitor treatment response (9, 10). The clinical challenge now lies in integrating imaging findings with serum markers and metabolic risk assessment to support management decisions (11, 12). We therefore focus on diagnostic performance, prognostic implications, and clinical integration of imaging-based assessment in MASLD, with the aim of developing an actionable framework that links pathophysiology, imaging biomarkers, risk modifiers, and management consequences.

## 2 Literature search and evidence selection

This narrative review was based on a structured search of the literature to make the evidence selection process explicit. PubMed/MEDLINE, Embase, Web of Science, and the Cochrane Library were searched from database inception to May 2026. The search used combinations of terms related to disease nomenclature, fibrosis assessment, and imaging-based or serum-based non-invasive tests, including “MASLD,” “NAFLD,” “MASH,” “NASH,” “fibrosis,” “liver stiffness,” “elastography,” “vibration-controlled transient elastography,” “VCTE,” “FibroScan,” “point shear wave elastography,” “pSWE,” “two-dimensional shear wave elastography,” “2D-SWE,” “magnetic resonance elastography,” “MRE,” “MRI-PDFF,” “corrected T1,” “cT1,” “multiparametric MRI,” “ELF,” and “FIB-4.” We primarily considered English-language articles. Non-English articles with English abstracts were screened when identified, but studies without sufficient English-language information for interpretation were not prioritized. Reference lists of relevant guidelines and major reviews were also checked for additional key studies. Because much of the evidence was published before the MASLD/MASH nomenclature was introduced, studies using NAFLD/NASH terminology were considered relevant when their populations were consistent with current metabolic dysfunction-associated steatotic liver disease concepts (13). Evidence was prioritized in the following order: international guidelines, systematic reviews and meta-analyses, prospective cohorts, biopsy-referenced diagnostic studies, longitudinal outcome studies, and studies directly addressing clinical pathways or treatment monitoring. This review was not intended to be a systematic review; therefore, formal risk-of-bias assessment and quantitative evidence synthesis were not performed.

## 3 Why fibrosis assessment matters in MASLD

Fibrosis stage is the main determinant of prognosis in MASLD. Landmark longitudinal biopsy-based cohorts have consistently shown that fibrosis stage, rather than steatohepatitis activity alone, is the strongest histological predictor of liver-related outcomes and mortality. Compared with patients without fibrosis, liver-related mortality increases progressively with fibrosis severity (3, 14, 15). A large meta-analysis, as summarized in recent reviews and guidelines, reported pooled relative risks for liver-related mortality of approximately 4.1 for significant fibrosis at stage F2 or higher, 7.6 for advanced fibrosis at stage F3 or higher, and 15.1 for cirrhosis at stage F4 (16). Fibrosis staging is also relevant before cirrhosis develops. Many patients with MASLD have normal or only mildly elevated aminotransferase levels, and routine laboratory testing may underestimate disease severity. Accordingly, contemporary guidance recommends structured risk stratification rather than reliance on liver enzymes alone (17).

Risk stratification in MASLD is not confined to liver-specific outcomes. Fibrosis severity has also been associated with cardiovascular disease, chronic kidney disease, and overall mortality (18). This broader prognostic relevance reflects the close relationship between MASLD, cardiovascular disease, chronic kidney disease, and systemic metabolic dysfunction. Imaging is therefore valuable not only because it estimates fibrosis stage, but also because it helps place the liver within a larger risk profile. These clinical demands favor tests that can be repeated, interpreted in context, and incorporated into sequential pathways (19, 20). A practical strategy is to use complementary tests to first exclude advanced disease, then refine staging in patients who remain uncertain or clinically high risk.

## 4 Pathophysiological basis of imaging in fibrosis

Fibrosis changes the physical behavior of the liver. In MASLD, progressive extracellular matrix deposition, architectural distortion, and altered intrahepatic vascular resistance increase liver stiffness and alter tissue mechanics (21–23). Ultrasound-based and magnetic resonance-based elastography techniques exploit these biomechanical changes to estimate liver stiffness non-invasively (24). Stiffness, however, is not specific to fibrosis alone. Inflammation, congestion, cholestasis, and postprandial state may increase measured stiffness and confound interpretation (21). This limitation applies broadly to stiffness-based assessment and should be considered during interpretation, particularly in patients with possible inflammatory, cholestatic, or hemodynamic confounders (21, 25).

Fibrosis is also associated with changes in tissue composition and perfusion. Quantitative magnetic resonance imaging (MRI) techniques can capture some of these features (10, 26, 27). Proton density fat fraction measures hepatic fat content with high reproducibility, whereas T1-based approaches may reflect fibroinflammatory change (28, 29). Perfusion-related and vascular changes become increasingly relevant as disease progresses toward portal hypertension and decompensation (30–32).

**Figure 1:**
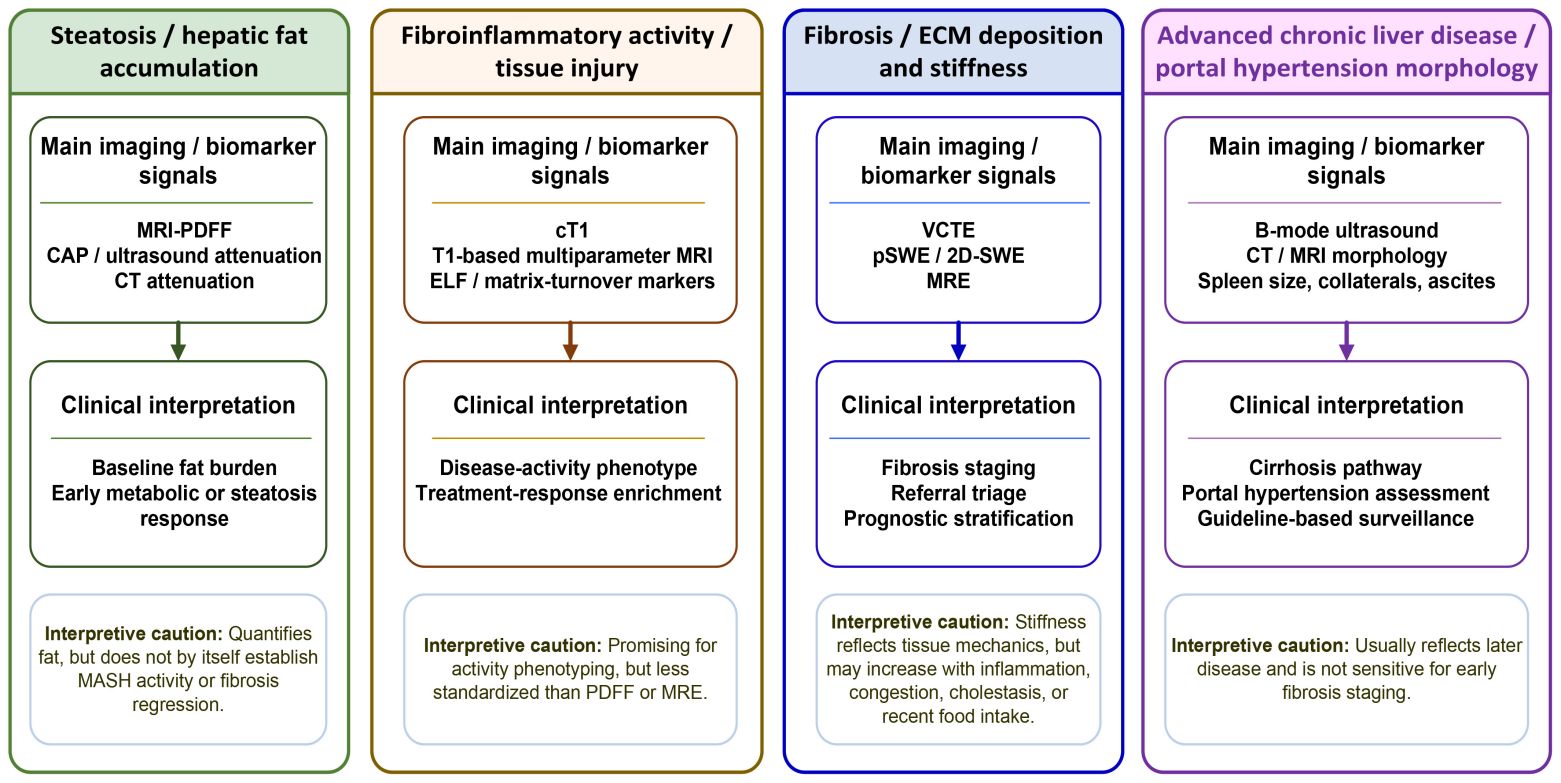
Pathophysiological domains and complementary imaging biomarkers in MASLD. The figure links major biological domains in MASLD with imaging and biomarker signals that are commonly used for disease characterization. MRI-PDFF, CAP, and CT attenuation primarily reflect hepatic fat accumulation and are most useful for quantifying steatosis or early metabolic response. cT1, multiparametric MRI, and ELF may provide information related to fibroinflammatory activity or matrix turnover, although these markers are less standardized than PDFF or MRE. VCTE, pSWE, 2D-SWE, and MRE assess liver stiffness as a mechanical correlate of fibrosis and extracellular matrix remodeling, but stiffness values may also be influenced by inflammation, cholestasis, congestion, and recent food intake. B-mode ultrasound, CT/MRI morphology, spleen size, collateral vessels, and ascites mainly reflect later-stage chronic liver disease or portal hypertension morphology rather than early fibrosis. These biomarkers should therefore be interpreted as complementary signals that answer different clinical questions, rather than as interchangeable measures of a single pathological process. CAP, controlled attenuation parameter; CT, computed tomography; cT1, corrected T1; ELF, enhanced liver fibrosis test; ECM, extracellular matrix; MASH, metabolic dysfunction-associated steatohepatitis; MASLD, metabolic dysfunction-associated steatotic liver disease; MRE, magnetic resonance elastography; MRI, magnetic resonance imaging; MRI-PDFF, magnetic resonance imaging proton density fat fraction; PDFF, proton density fat fraction; pSWE, point shear wave elastography; 2D-SWE, two-dimensional shear wave elastography; VCTE, vibration-controlled transient elastography.

A complete assessment may combine indicators of fibrosis, steatosis, inflammatory activity, and portal hypertension (33, 34). Because MASLD involves fibrosis, steatosis, fibroinflammatory activity, and hemodynamic alteration simultaneously, imaging assessment increasingly relies on multiple complementary biomarkers rather than stiffness alone. Figure 1 summarizes how extracellular matrix deposition, fibroinflammatory activity, steatosis, and hemodynamic alteration correspond to different imaging biomarkers. Table 1 summarizes the principal imaging biomarkers, their biological correlates, and their major clinical applications.

**Table 1:** Key imaging biomarkers in MASLD and their pathophysiological correlates and clinical applications.

Biomarker	Imaging modality	Pathophysiological domain	Reflects	Clinical application
Liver stiffness	US elastography (VCTE, SWE); MRE	Tissue stiffness	Fibrosis severity (ECM deposition)	Fibrosis staging; risk stratification; prediction of liver-related outcomes
Proton density fat fraction (PDFF)	MRI	Tissue composition	Hepatic steatosis (fat accumulation)	Quantification of steatosis; monitoring treatment response
T1 mapping/corrected T1 (cT1)	MRI	Tissue composition	Inflammation and fibrosis-related changes	Disease activity assessment; prediction of disease progression
Perfusion parameters	Contrast-enhanced imaging; perfusion MRI/CT	Hemodynamic alterations	Intrahepatic vascular resistance and perfusion changes	Evaluation of advanced disease; emerging role in risk stratification
Multiparametric MRI (combined biomarkers)	MRI	Integrated (stiffness + composition)	Fibrosis, steatosis, and inflammation	Comprehensive disease assessment; longitudinal monitoring; clinical trials

MASLD, metabolic dysfunction-associated steatotic liver disease; US, ultrasound; VCTE, vibration-controlled transient elastography; SWE, shear wave elastography; MRE, magnetic resonance elastography; cT1, corrected T1; MRI, magnetic resonance imaging; ECM, extracellular matrix.

## 5 Ultrasound-based imaging techniques

Ultrasound-based techniques are usually the initial imaging tools for fibrosis assessment in MASLD pathways. Their appeal is practical: they are widely available, comparatively inexpensive, rapid to perform, and easy to integrate into stepwise assessment strategies recommended by recent AASLD and EASL guidance (35–37).

Conventional B-mode ultrasound has limited sensitivity for early fibrosis. Surface nodularity, coarsened echotexture, widened fissures, and secondary signs of portal hypertension are usually late findings and therefore do not support reliable staging of earlier disease (38). Its main value is opportunistic detection of steatosis and recognition of overt chronic liver disease rather than fibrosis quantification (39, 40).

Elastography has changed the role of ultrasound by providing quantitative liver stiffness measurement (41–43). The major methods used in MASLD are vibration-controlled transient elastography, point shear wave elastography, and two-dimensional shear wave elastography. These techniques differ in implementation, but they share a common clinical purpose: triage patients more accurately than routine biochemistry or conventional ultrasound alone (44–47).

### 5.1 Vibration-controlled transient elastography

Vibration-controlled transient elastography (VCTE) remains the most established ultrasound-based method in MASLD, supported by early validation studies and subsequent guideline recommendations (48, 49). In a US biopsy-referenced NAFLD cohort, Tapper et al. (49) showed that VCTE had clinically useful performance for detecting advanced fibrosis, reinforcing its role as a practical frontline triage tool. Performance is generally strongest for advanced fibrosis and cirrhosis. In a recent MASLD-specific meta-analysis, pooled performance for advanced fibrosis (stage F3 or higher) reached an AUC of 0.84 with sensitivity 88.1% and specificity 63.8%, whereas performance for cirrhosis reached an AUC of 0.90 with sensitivity 76.5% and specificity 87.7% (50). Accordingly, VCTE performs best for excluding or identifying advanced fibrosis rather than separating adjacent early fibrosis stages.

VCTE results should not be interpreted solely according to fixed cutoffs; instead, they should be considered in relation to the patient's pre-test risk, clinical context, and measurement reliability (51). Values below commonly used rule-out ranges support reassurance in low-prevalence settings, whereas clearly elevated values raise concern for compensated advanced chronic liver disease and justify escalation. Intermediate results should not be overinterpreted. They are best understood in relation to pre-test risk, platelet count, aminotransferases, diabetes status, and whether the acquisition was technically reliable (52).

Several factors can shift VCTE upward independent of fibrosis. Active necroinflammation, cholestasis, hepatic congestion, postprandial state, and technical limitations related to obesity or narrow intercostal spaces may all affect results (25, 53). The XL probe has improved feasibility in patients with BMI of 30 kg/m^2^ or greater, but severe obesity still reduces confidence in some cases (54–56). Reliability is also influenced by fasting status, operator technique, and whether the measurement quality criteria are satisfied (12, 52, 57–59).

These limitations are most relevant in technically difficult examinations and in patients with borderline stiffness values. VCTE is best viewed as a scalable first-line imaging test that efficiently separates patients with low near-term likelihood of advanced fibrosis from those who need confirmation, closer follow-up, or referral. It becomes less useful when asked to resolve biologically complex borderline cases on its own (48, 49, 60). Because VCTE is widely scalable, there is also a risk that single cutoff values may be overapplied without sufficient attention to disease prevalence, metabolic risk burden, or acquisition quality (12, 52, 61).

In clinical practice, VCTE-derived liver stiffness should be interpreted as an approximate risk category rather than a fixed diagnostic boundary. Commonly used thresholds in MASLD include values below approximately 8 kPa to indicate a low likelihood of advanced fibrosis, values around 8–12 kPa as an indeterminate range, and values above approximately 12–15 kPa as raising concern for advanced fibrosis or compensated advanced chronic liver disease. Higher values, particularly in the range of 20–25 kPa or above, should prompt consideration of cirrhosis-related pathways and portal hypertension risk stratification, especially when combined with platelet count and other clinical features (62). These thresholds should be interpreted cautiously in patients with obesity, type 2 diabetes, ALT flare, cholestasis, hepatic congestion, recent food intake, or technically limited examinations. Table 2 summarizes commonly used VCTE interpretive categories, suggested clinical actions, and key quality considerations (6, 51, 62–64).

**Table 2:** Practical interpretation of VCTE-derived liver stiffness in MASLD.

VCTE liver stiffness range	Practical interpretation	Suggested clinical action	Major caveats
< 8 kPa	Low likelihood of advanced fibrosis	Follow in primary/secondary care; repeat risk assessment according to metabolic risk	Does not fully exclude disease in high-risk patients, especially T2D, obesity, or discordant serum/imaging findings
8–12 kPa	Indeterminate/possible fibrotic MASH or significant fibrosis	Check quality criteria and confounders; consider ELF, MRE, or hepatology referral if clinically indicated	ALT flare, cholestasis, congestion, recent food intake, and obesity may increase stiffness
>12–15 kPa	Concern for advanced fibrosis/cACLD	Hepatology referral; consider MRE or biopsy if results are discordant or management-changing	Thresholds are not universal and should be interpreted with clinical context
≥20–25 kPa	Concern for cirrhosis and/or portal hypertension risk	Evaluate for cirrhosis-related management, portal hypertension risk stratification, and surveillance pathways	Platelet count and Baveno criteria should be integrated; stiffness alone should not define portal hypertension

ALT, alanine aminotransferase; cACLD, compensated advanced chronic liver disease; MASLD, metabolic dysfunction-associated steatotic liver disease; MASH, metabolic dysfunction-associated steatohepatitis; MRE, magnetic resonance elastography; T2D, type 2 diabetes; VCTE, vibration-controlled transient elastography.

Reliable VCTE interpretation also requires attention to examination quality. In general, the report should include the probe type, fasting status, number of valid measurements, median liver stiffness, and IQR/median ratio. An examination based on at least 10 valid measurements and an IQR/median ratio of 30% or lower is commonly considered technically reliable, although interpretation should still consider the absolute stiffness value and clinical context. Patients with obesity may require the XL probe, and failed or unreliable measurements should prompt repeat examination under optimized conditions or use of alternative methods such as SWE, ELF, or MRE. For longitudinal follow-up, serial liver stiffness values are most meaningful when obtained using the same platform, comparable fasting conditions, appropriate probe selection, and similar acquisition standards (12, 52, 57–59).

### 5.2 Point shear wave and two-dimensional shear wave elastography

Not all ultrasound elastography is blind or stand-alone. Point shear wave elastography (pSWE) and two-dimensional shear wave elastography (2D-SWE) extend stiffness measurement into a conventional ultrasound examination (65, 66). Early biopsy-referenced validation work, including the study by Ferraioli et al. (67), established pSWE as a feasible method for liver stiffness assessment before later meta-analytic evidence consolidated its diagnostic role. Their practical advantage is direct B-mode guidance. The operator can choose a measurement window, avoid large vessels or focal lesions, and evaluate morphology in the same sitting. This is especially useful in centers that already rely on diagnostic ultrasound rather than a separate FibroScan pathway (45, 67, 68).

Point shear wave elastography has been validated in biopsy-referenced studies and generally shows diagnostic performance comparable to VCTE when acquisition is standardized (67). In a recent meta-analysis of MASLD cohorts, pooled AUROCs for advanced fibrosis (*F* ≥ 3) were 0.912 for pSWE and 0.85 for 2D-SWE (69). The main challenge is the limited transferability of cutoffs across vendors and platforms. Vendor-specific implementations differ in acoustic radiation force generation, display format, quality metrics, and reporting units, which means one platform's threshold cannot simply be transplanted to another (70–73). Quality control therefore matters more for shear wave methods than many clinicians appreciate. Probe pressure, region-of-interest placement, breath-hold quality, depth of sampling, and avoidance of reverberation or subcapsular artifacts all influence the final value. Obesity, inflammation, congestion, and cholestasis remain relevant confounders, just as with VCTE (74–76). In follow-up settings, serial comparison is most meaningful when the same platform, protocol, and operator standard are maintained.

In clinical practice, pSWE and 2D-SWE are most useful when integrated ultrasound assessment and direct B-mode guidance improve acquisition confidence (69, 77, 78). Their limitation is not low biological relevance but lower standardization across vendors and sites. Accordingly, these methods are best interpreted within local expertise and technique-specific protocols rather than with universal numeric cutoffs (69, 79, 80). Historically, other ultrasound elastography approaches, including real-time tissue elastography and acoustic radiation force impulse-based implementations, have also been investigated (81–83). However, current MASLD-oriented clinical frameworks primarily focus on VCTE, pSWE, and 2D-SWE because these methods have the strongest diagnostic evidence and the clearest relevance to contemporary clinical pathways (69, 84).

A further standardization issue is that VCTE, pSWE, and 2D-SWE should not be treated as interchangeable methods simply because they all estimate liver stiffness (69, 72, 73, 80, 85). VCTE is a dedicated non-imaging examination, whereas pSWE and 2D-SWE are performed within a conventional ultrasound examination and allow B-mode-guided measurement (65–67). In pSWE, the measurement is obtained from a focal region of interest at a selected depth; in 2D-SWE, the operator selects regions of interest from a two-dimensional stiffness map (67, 73, 78, 80). Reported values may be expressed in kilopascals or as shear-wave speed in meters per second, but conversion between these units assumes simplified tissue properties and may not be equivalent across vendors, acquisition settings, or disease states (70, 72, 73). Depth of sampling, region-of-interest placement, subcapsular artifacts, rib shadowing, probe pressure, and breath-hold quality can all affect the final value (73, 76, 85). For this reason, pSWE and 2D-SWE are best considered locally validated alternatives or complements to VCTE rather than universally equivalent substitutes in clinical pathways (47, 77, 78, 80, 85). These practical differences are summarized in Table 3.

**Table 3:** Practical comparison of VCTE, pSWE, and 2D-SWE in MASLD.

Feature	VCTE	pSWE	2D-SWE
Acquisition method	Dedicated non-imaging elastography examination	Integrated into conventional ultrasound	Integrated into conventional ultrasound with 2D stiffness map
Imaging guidance	No real-time B-mode guidance for ROI selection	B-mode-guided focal ROI	B-mode-guided elastographic map with selectable ROI
Reported unit	Usually kPa	m/s or kPa depending on vendor	m/s or kPa depending on vendor
Reliability considerations	Valid measurements, IQR/median, fasting status, probe selection	Depth, ROI placement, breath-hold, artifacts, vendor-specific quality indicators	Map quality, ROI placement, depth, artifacts, vendor-specific quality indicators
Vendor dependence	Lower than pSWE/2D-SWE but still present	Important	Important
Clinical role	Most established frontline pathway tool	Locally validated alternative/complement	Locally validated alternative/complement
Main caution	Cutoffs should not be overapplied across populations	Cutoffs are not freely transferable across platforms	Cutoffs are not freely transferable across platforms

2D-SWE, two-dimensional shear wave elastography; IQR, interquartile range; kPa, kilopascal; MASLD, metabolic dysfunction-associated steatotic liver disease; m/s, meters per second; pSWE, point shear wave elastography; ROI, region of interest; VCTE, vibration-controlled transient elastography.

Standardization of shear-wave speed measurement remains an active area of work rather than a solved problem. RSNA QIBA has proposed a shear-wave speed profile for liver fibrosis assessment, and WFUMB/EFSUMB and Society of Radiologists in Ultrasound guidance documents similarly emphasize standardized acquisition, quality control, reporting, and cautious use of vendor-specific thresholds (86–88). Cross-platform studies have also shown that technical success, reliability, and stiffness values may differ across pSWE and 2D-SWE systems (89). In practice, pSWE and 2D-SWE cutoffs should not be transferred directly across manufacturers or software versions. Serial follow-up should use the same vendor, platform, probe, acquisition depth, ROI strategy, reporting unit, and quality criteria whenever possible; if a different system is used, clinicians should interpret changes as broad risk-category shifts rather than precise numerical evidence of fibrosis progression or regression, and should confirm discordant or management-changing results with VCTE, ELF, or MRE (86–88).

### 5.3 Clinical use of ultrasound elastography

Ultrasound elastography is most useful when framed as a decision-support test rather than as a stand-alone diagnostic endpoint (90–92). Its main role is to identify patients who require closer evaluation or specialist referral (79, 93). Another practical advantage is that ultrasound-based stiffness measurement can be repeated in routine care, combined with standard abdominal ultrasound, and deployed in settings where MRI is not immediately available. This is especially relevant in broad metabolic populations, where the challenge is large-scale triage rather than confirmation of rare liver disease (94, 95).

Borderline stiffness values are most informative when read alongside measurement reliability, platelet count, aminotransferases, metabolic risk burden, and the plausibility of confounders such as congestion or inflammation (93, 96–98). A technically acceptable low result may support reassurance; an equivocal result should usually prompt repetition, contextual reinterpretation, or a more definitive second-line test rather than immediate overclassification (99).

Ultrasound elastography also has a role in longitudinal care, but serial interpretation is meaningful only when acquisition is methodologically consistent. Values are more interpretable when the same platform, probe strategy, fasting conditions, and acquisition quality standards are maintained (100, 101). The clinical question during follow-up is usually whether the patient's risk profile is changing in a meaningful and persistent way, rather than whether small numerical shifts are sufficient to reassign a patient from one histologic stage to another (100, 102). Ultrasound elastography therefore serves as the practical bridge between serum-based screening and selective specialist evaluation. It makes fibrosis triage scalable, while preserving a role for more definitive tools when uncertainty remains (93, 103).

## 6 Magnetic resonance imaging techniques

MRI-based techniques occupy the next tier of non-invasive assessment in MASLD. They are more resource-intensive than ultrasound, but they provide broader tissue characterization, lower operator dependence, and better performance in clinical scenarios where ultrasound is unreliable or incomplete (104–108).

MRI can assess fibrosis directly through elastography and can simultaneously characterize steatosis and other tissue properties through quantitative methods (29, 109–111). This multiparametric capability is useful when the clinical question extends beyond whether advanced fibrosis is present and begins to include disease activity, baseline characterization before therapy, or interpretation of discordant frontline tests (112–114).

### 6.1 Magnetic resonance elastography (MRE)

Magnetic resonance elastography (MRE) occupies a different tier of care. Seminal validation studies, including the early work by Yin et al. (115), established the diagnostic utility of MRE for hepatic fibrosis assessment. In MASLD, its value is greatest when ultrasound-based assessment is technically unreliable, discordant with clinical risk, or insufficiently precise for management decisions (107, 115–117). Its advantage comes from both higher diagnostic performance and more stable whole-liver sampling (85, 107, 118).

In current specialist practice, MRE is often regarded as the most accurate imaging-based non-invasive test for fibrosis staging in MASLD, a position reinforced by individual-patient meta-analyses and subsequent diagnostic studies (108, 119). In an individual-patient meta-analysis, validated MRE cutoffs were 3.14 kPa for significant fibrosis (F2 or higher), 3.53 kPa for advanced fibrosis (F3 or higher), and 4.45 kPa for F4 (120). These cutoffs are most useful in patients with discordant frontline tests, technically unreliable ultrasound examinations, or high-risk metabolic profiles (108, 120). Even for MRE, evidence is strongest for advanced fibrosis and cirrhosis, whereas discrimination around lower fibrosis thresholds may be more vulnerable to inflammatory activity, technical protocol differences, and histologic reference-standard limitations (118, 121–123).

MRE also provides more reproducible measurements in longitudinal follow-up, treatment trials, and specialist clinics where serial change may matter as much as a categorical fibrosis label (124–126). Whole-liver assessment also helps when disease distribution is heterogeneous, which is relevant in MASLD because fibrosis and steatosis are not always spatially uniform. In practice, MRE is most useful when frontline serum-based or ultrasound-based assessment remains indeterminate or technically unreliable (108, 127).

In practice, the main question is not whether MRE is more accurate, but whether the additional information is likely to change management. MRE is most useful after failed or unreliable VCTE, in patients with severe obesity and persistent clinical suspicion, or when FIB-4 and VCTE remain indeterminate or discordant (107, 108, 120, 127). It may also be appropriate when confirmation of advanced fibrosis would affect pharmacologic treatment decisions, clinical trial enrollment, selected pre-bariatric surgery assessment, or tertiary-care phenotyping (124–127). In these settings, the higher cost of MRE may be justified by improved diagnostic confidence, broader liver sampling, and reduced dependence on acoustic windows or operator technique (107, 108, 127). A practical framework for selecting patients in whom MRE may justify its cost and resource requirements is provided in Table 4.

**Table 4:** Practical indications and limitations of MRE in MASLD.

Use case	Rationale	Suggested role
Failed or unreliable VCTE	Obesity, narrow intercostal spaces, poor acoustic windows, or suboptimal acquisition may limit ultrasound-based stiffness measurement	Second-line test after technically unreliable ultrasound elastography
Severe obesity with high clinical suspicion	MRE is less dependent on acoustic windows and operator technique than ultrasound-based elastography	Clarify fibrosis risk when VCTE is unreliable or discordant with metabolic risk
Indeterminate FIB-4 and indeterminate VCTE	Sequential first-line testing may leave unresolved risk categories	Improve diagnostic confidence and guide referral or confirmatory evaluation
Discordant serum and imaging tests	Serum markers and stiffness-based imaging can be affected by different biological and technical confounders	Resolve clinically important discordance before escalation, surveillance planning, or biopsy decisions
Suspected advanced fibrosis before therapy	Treatment eligibility and monitoring intensity may depend on fibrosis severity	Confirm or refine fibrosis severity when the result may change management
Clinical trial screening	MRE provides quantitative and reproducible whole-liver stiffness assessment	Enrich trial populations and support longitudinal monitoring
Selected pre-bariatric surgery assessment	Severe obesity and metabolic risk may reduce VCTE reliability, while advanced fibrosis affects perioperative planning	Use selectively in high-risk candidates with uncertain fibrosis status
Tertiary-care phenotyping	Whole-liver stiffness assessment can complement MRI-based steatosis and tissue characterization	Support detailed specialist assessment when clinically actionable
Population screening	Cost, scanner availability, examination time, reimbursement, hardware/software needs, and radiology expertise limit scalability	Not suitable as first-line screening for unselected MASLD populations

FIB-4, fibrosis-4 index; MASLD, metabolic dysfunction-associated steatotic liver disease; MRE, magnetic resonance elastography; VCTE, vibration-controlled transient elastography.

These advantages do not make MRE a population-level screening tool. Wider use is constrained by scanner availability, examination time, cost, reimbursement uncertainty, dedicated hardware and post-processing requirements, and local radiology expertise (128, 129). Such constraints matter most in primary-care, diabetes, obesity, and metabolic clinics, where large numbers of at-risk individuals require triage. Technical failure can still occur with substantial iron overload, severe motion artifact, or limited local expertise (129, 130), and MRE values may be influenced by inflammatory activity, especially near lower fibrosis thresholds (131, 132). MRE is therefore best positioned as a selective second-line or tertiary-care test when additional diagnostic confidence is likely to affect referral, treatment eligibility, surveillance planning, trial enrollment, or biopsy decisions (6, 105, 120, 124).

### 6.2 Multiparametric MRI

Different MRI-derived biomarkers capture distinct biological features of MASLD, including stiffness, steatosis, and fibroinflammatory activity. MRE, magnetic resonance imaging proton density fat fraction (MRI-PDFF), and T1-based approaches describe different aspects of the liver: stiffness, fat burden, and fibroinflammatory activity (29, 133). Combined interpretation of these parameters may better characterize steatosis burden, fibroinflammatory activity, and structural remodeling.

Patients with similar stiffness values may still show different degrees of steatosis or fibroinflammatory activity. One patient may have marked steatosis with limited structural change, whereas another may have less fat but more active fibroinflammatory injury (134–136). Multiparametric MRI is most useful when fibrosis severity alone does not adequately describe disease phenotype.

MRI-PDFF is the most mature quantitative MRI biomarker for hepatic steatosis and has been extensively validated against histology and MR spectroscopy in foundational studies by Reeder et al. (137), Yokoo et al. (138), and Tang et al. (139). It provides highly reproducible quantification of hepatic steatosis, with current guidance generally treating values of 5% or greater as consistent with steatosis (140–143). In the exemplar imaging review and related studies, MRI-PDFF showed sensitivity ranging from 77% to 92% and specificity ranging from 87% to 94% for any steatosis, with AUROCs of 0.98 for any steatosis, 0.91 for moderate steatosis, and 0.92 for severe steatosis (10, 27). PDFF is particularly useful for establishing quantitative baseline steatosis burden and tracking longitudinal change over time.

A practical distinction is needed between reduction in liver fat, improvement in steatohepatitis activity, and true fibrosis regression (27, 144, 145). PDFF is best understood as a quantitative marker of steatosis and as a useful enrichment or response biomarker in trials, rather than as a stand-alone marker of matrix remodeling or long-term liver-related risk (27, 137–145). Liver fat may fall relatively quickly after weight loss, GLP-1 receptor agonist therapy, or other metabolic interventions, but inflammatory activity and fibrosis usually change on a different timescale (145–147). In routine care, PDFF improvement alone should not be used to infer fibrosis regression unless supported by stiffness-based imaging, serum markers, histology, or longitudinal clinical assessment (144, 145).

During disease progression or treatment response, these MRI-derived signals do not necessarily change in parallel (144, 145). Liver fat may improve rapidly after weight loss or metabolic therapy, whereas stiffness and fibroinflammatory markers may evolve more slowly (144–146). Combined MRI assessment can therefore help distinguish early metabolic response from slower structural remodeling, which is often more informative than relying on one parameter alone.

This has practical implications for treatment planning and trial design. Before therapy, multiparametric MRI can provide a cleaner baseline phenotype than routine laboratory testing alone. During follow-up, it can show whether apparent improvement is confined to steatosis reduction or is accompanied by broader change in disease activity (148–150). In selected tertiary-care patients, it may also help reconcile discordant results from serum markers and elastography by showing that the dominant abnormality is fat, fibroinflammation, or advanced structural disease (129, 151, 152).

Despite its biological richness, multiparametric MRI remains difficult to standardize fully across platforms and acquisition pipelines. Acquisition protocols, post-processing pipelines, and threshold definitions are not fully harmonized, and some biomarkers remain more vendor-dependent than MRE (153). Multiparametric MRI is therefore most valuable in specialist assessment, treatment monitoring, and research settings where its additional information is likely to change interpretation rather than simply duplicate what frontline tests have already shown. In particular, corrected T1 (cT1) and other fibroinflammatory markers remain promising but are less uniformly standardized than PDFF or MRE, and their incremental value over stiffness-based assessment may depend on the clinical setting and outcome being predicted (149, 150, 154).

### 6.3 MRI in clinical pathways

MRI is usually introduced after initial serum-based and ultrasound-based evaluation, particularly when elastography is indeterminate, technically unreliable, or discordant with the broader clinical picture (152, 155). It is also useful when simultaneous characterization of fibrosis and steatosis is needed, especially before therapy or in trial settings where quantitative reproducibility is important (156, 157). MRI therefore occupies a selective second-line role in MASLD, particularly when greater diagnostic confidence is required.

## 7 Computed tomography (CT) and selected emerging tools

CT is not a primary fibrosis-staging tool in MASLD. Its role is mainly adjunctive or opportunistic. Conventional CT can detect surface nodularity, altered lobar morphology, widened fissures, collateral vessels, splenomegaly, and other signs associated with advanced chronic liver disease, but it lacks sensitivity for early fibrosis and is not suited to repeated monitoring because of radiation exposure (158–161).

Quantitative CT-based approaches, including texture analysis, extracellular volume estimation, and liver surface nodularity metrics, have been explored as ways to extract more information from routine imaging (159, 160). These tools are interesting, but their performance remains less mature than VCTE or MRE and they have not entered standard first-line pathways (161).

Radiomics and artificial intelligence are promising but remain immature for routine MASLD fibrosis assessment. Liver-specific studies suggest that quantitative features extracted from ultrasound, CT, or MRI may improve automated fibrosis classification or multimodal risk prediction, especially when combined with clinical and laboratory variables (162–165). However, much of the evidence remains retrospective, single-center, and based on curated datasets. Model performance may fall when applied across different scanners, vendors, acquisition protocols, segmentation methods, and patient populations. External validation, calibration, decision-curve analysis, and prospective workflow testing are therefore more important than reporting AUROC alone (163, 166).

At present, AI-based tools should be regarded as emerging adjuncts rather than replacements for established serum-based tests, elastography, or MRI-based assessment (166, 167). Before clinical adoption, models need interpretable outputs, reproducible segmentation or automated quality control, evaluation in real-world MASLD populations, and evidence that they improve clinical decisions rather than only diagnostic classification metrics. Integration with electronic health records and laboratory data may eventually support risk-stratified pathways, but this remains a development goal rather than a standard component of current MASLD care (166, 168).

## 8 Comparative performance and modality selection

In MASLD, imaging modalities are usually selected according to clinical context rather than diagnostic accuracy alone. In real-world MASLD care, ultrasound elastography and MRE usually serve complementary rather than competing clinical roles. Ultrasound-based methods provide scalable frontline assessment, whereas MRE is reserved for cases where a more reproducible and comprehensive evaluation is needed (169).

From a diagnostic standpoint, MRE consistently outperforms ultrasound-based techniques across fibrosis thresholds, especially in obesity and in earlier disease where misclassification matters (118, 120, 170). By contrast, ultrasound elastography offers lower cost and broader availability, which makes it the more realistic population-level tool despite lower specificity in some settings (1, 6, 171).

Reproducibility also shapes modality selection. MRE has an advantage when serial measurements are required, such as in clinical trials or in patients whose management may depend on small but meaningful quantitative change (131, 172, 173). Ultrasound-based techniques remain useful in follow-up, but longitudinal interpretation must be more cautious because operator and platform effects are harder to eliminate (174).

Cost and access remain decisive. The most effective strategy is therefore sequential rather than competitive: serum-based triage first, ultrasound elastography second, and MRI selectively for unresolved or high-stakes questions (108, 111, 175–179). Supplementary Table S1 provides a practical comparison of the main imaging modalities, including diagnostic role, approximate interpretive ranges, reliability considerations, cost/access, reproducibility, monitoring utility, and best-fit clinical setting.

## 9 Imaging for risk stratification and prognosis

Imaging findings increasingly contribute to prognosis assessment in MASLD, not only fibrosis staging. Liver stiffness behaves as a continuous risk marker, not simply as a binary fibrosis category. Longitudinal studies of non-invasive tests have further shown that liver stiffness-based assessment can stratify patients according to their subsequent risk of liver-related events, extending the role of elastography beyond fibrosis staging toward outcome-oriented risk prediction (58, 171, 180). Fibrosis stage remains the main determinant of liver-related outcomes, but imaging converts that concept into a quantitative framework. Liver stiffness is a continuous variable, and risk rises along a gradient rather than appearing abruptly at a single histologic boundary (181). This makes imaging particularly useful for estimating monitoring intensity, especially in patients whose risk cannot be captured adequately by a binary fibrosis classification. Liver-related outcomes are the clearest example. Higher stiffness values are associated with progression to cirrhosis, hepatic decompensation, liver-related death, and incident hepatocellular carcinoma (182–184). In a meta-analysis of 1,707 patients with MASLD followed for a median of 3 years, MRE values between 5 and 8 kPa were associated with a hazard ratio of 11.0 for liver-related outcomes, while values of 8 kPa or higher were associated with a hazard ratio of 15.9 (185). These findings support interpreting stiffness as a prognostic gradient rather than only as a staging threshold. These data are clinically compelling, but they should be interpreted with attention to cohort composition, referral setting, and competing cardiometabolic risks, because prognostic thresholds derived from enriched cohorts may not transfer directly to lower-prevalence primary-care populations (186, 187).

Hepatocellular carcinoma (HCC) risk adds another layer. Surveillance recommendations still focus on advanced fibrosis and cirrhosis, yet MASLD-related HCC can develop before overt cirrhosis is clinically recognized (188–190). Imaging is therefore useful because it identifies patients whose risk appears higher than routine biochemical testing suggests. In the same MRE outcome literature, the 3-year cumulative HCC incidence was 0.35% in patients with MRE values below 5 kPa compared with 5.25% in those with values between 5 and 8 kPa and 5.66% in those with values of 8 kPa or higher (185).

These numbers should be used for risk refinement, not as a new surveillance rule. Higher stiffness may identify patients who deserve closer clinical attention, but it should not by itself create an indication for HCC surveillance outside guideline-supported settings such as advanced fibrosis or cirrhosis (188–190). Non-cirrhotic MASLD-related HCC remains an important clinical problem, but current evidence is not sufficient to define stiffness-only surveillance thresholds for broad MASLD populations (185, 191). This is particularly relevant when thresholds come from referral or enriched cohorts rather than primary-care, diabetes-clinic, or obesity-clinic populations (186, 187).

Prognostic value is not confined to liver-specific events. Increasing fibrosis severity in MASLD has also been associated with cardiovascular outcomes and all-cause mortality, reflecting the systemic metabolic context in which liver disease develops (192, 193). For this reason, imaging helps place hepatic fibrosis within a broader risk profile rather than treating the liver as an isolated organ. Elevated stiffness may therefore identify patients who are closer to cirrhosis and who also carry greater overall cardiometabolic vulnerability. A further advantage of imaging is that it can be repeated. Serial stiffness or quantitative MRI trajectories may eventually prove more informative than one baseline category, especially for identifying progression despite apparently stable routine laboratory values (194). Longitudinal imaging assessment remains less standardized than baseline staging, particularly regarding the degree of change that reflects clinically meaningful improvement.

## 10 Imaging for treatment monitoring

Imaging is increasingly being incorporated into longitudinal treatment assessment in MASLD. As pharmacologic options expand and structured weight-loss interventions become more common, clinicians need repeatable biomarkers that can separate early metabolic response from slower structural change (28, 110).

PDFF has become a widely used quantitative endpoint in clinical research because changes in liver fat can be detected reliably over time (195, 196). Loomba and colleagues helped establish MRI-PDFF as a treatment-response biomarker in NASH trials, and subsequent multicenter validation and meta-analytic evidence showed that a relative decline of at least 30% in MRI-PDFF is associated with histologic response, including steatohepatitis resolution and improvement in the NAFLD activity score (NAS) (27, 144). Consequently, MRI-PDFF is now widely used as a quantitative endpoint in proof-of-concept and phase 2 studies (197). Nevertheless, this threshold should be interpreted as evidence of steatosis reduction and biological response rather than as a standalone surrogate for fibrosis regression or long-term liver-related outcomes (27, 144, 145).

This distinction is important in therapeutic trials. A rapid fall in MRI-PDFF may show target engagement, weight-loss response, or early metabolic improvement, but it does not prove that advanced architectural fibrosis has resolved (27, 144, 145). The phase 2 semaglutide trial is a useful example: steatohepatitis resolution improved, but fibrosis improvement did not differ significantly from placebo (147). More recent tirzepatide and resmetirom trials have also retained histologic endpoints, including MASH resolution and fibrosis improvement, rather than relying on liver fat reduction alone (198, 199). These trial designs reflect a practical concern in MASLD: liver fat, inflammation, and matrix remodeling may improve on different timelines. MRI-PDFF is therefore best interpreted as one component of a multimodal endpoint framework, alongside stiffness-based imaging, serum fibrosis markers, histology when required, and ultimately clinical outcomes (27, 144, 145, 187, 200).

The expected imaging response depends on the intervention being monitored. Lifestyle-induced weight loss, bariatric surgery, GLP-1 receptor agonists, and dual or triple incretin-based therapies may produce relatively early reductions in liver fat, making MRI-PDFF useful for documenting steatosis response (27, 198, 201, 202). Thyroid hormone receptor-β agonists and other MASH-directed pharmacotherapies may also reduce liver fat, improve biochemical markers, and achieve histologic responses, although durable fibrosis improvement and long-term clinical benefit still require longer follow-up (199). Follow-up imaging should therefore distinguish early metabolic or steatosis response from slower changes in inflammatory activity and matrix remodeling (27, 144, 145).

The interpretation of clinically meaningful change also differs by biomarker. For MRI-PDFF, a relative reduction of at least 30% is commonly used in trials as an imaging signal of steatosis reduction and biological response (27, 144, 145). For liver stiffness measured by VCTE or MRE, clinically meaningful change is less standardized (186, 187, 203). Small stiffness changes may reflect measurement variability, fasting status, inflammatory activity, congestion, or acquisition differences rather than true fibrosis remodeling (12, 57–59, 131, 187). Serial stiffness should therefore be interpreted in relation to baseline fibrosis risk, measurement quality, platform consistency, serum marker trends, and whether the change is large and persistent enough to alter management (186, 187, 203).

The optimal interval for repeat imaging remains uncertain and should be individualized according to baseline fibrosis risk, treatment type, expected biological time course, and whether the result will influence management (58, 171, 180, 187). Serial VCTE is usually the most practical option for routine follow-up because it is scalable, repeatable, and less costly than MRI (1, 6, 171, 180). MRE or MRI-PDFF may be preferred in selected settings, including clinical trials, technically unreliable ultrasound examinations, discordant serum and elastography results, severe obesity, or treatment decisions that depend on more reproducible quantification of steatosis or stiffness (108, 120, 125, 144, 195). Imaging changes are most informative when linked to histologic, biochemical, or outcome-based anchors rather than interpreted as isolated numerical shifts (27, 144, 145, 187, 200).

## 11 Integration with serum-based non-invasive tests

In routine MASLD care, imaging and serum-based tests should not be treated as interchangeable. Serum-based tests and imaging biomarkers reflect different biological dimensions of disease. Serum-based markers mainly estimate fibrosis probability or matrix activity, whereas imaging reflects structural and mechanical consequences of liver injury (203). Combining them improves interpretation because MASLD severity is biologically heterogeneous rather than reducible to a single signal.

FIB-4 remains the most practical entry marker because it uses routinely available variables and performs well for broad risk exclusion in low-prevalence settings (204, 205). Its limitation is familiar: a substantial proportion of patients fall into an intermediate range, and age or metabolic comorbidity can shift interpretation (206, 207). FIB-4 is therefore best understood as a triage marker of probability, not as a stand-alone descriptor of liver phenotype.

ELF contributes different information. By capturing extracellular matrix remodeling and fibrogenic activity, it may provide a biological signal that is not fully represented by aminotransferases or platelet-based indices (208, 209). This makes ELF particularly useful when routine scores appear deceptively reassuring, when fibrosis risk seems out of proportion to standard laboratory testing, or when the clinician wants evidence of active fibrogenesis rather than only structural consequence.

Composite models illustrate how different non-invasive signals can be combined for risk enrichment. The FAST score was originally derived to identify patients with at-risk NASH by combining FibroScan-based liver stiffness, controlled attenuation parameter, and AST, whereas the MEFIB index combines FIB-4 with MRE to enrich for patients with greater fibrotic burden and higher-risk disease. Their clinical value lies in risk enrichment rather than universal screening. Therefore, FAST and MEFIB should be applied in the populations and decision settings for which they were developed, rather than used as interchangeable pathway tools across all MASLD settings (210–212).

**Figure 2:**
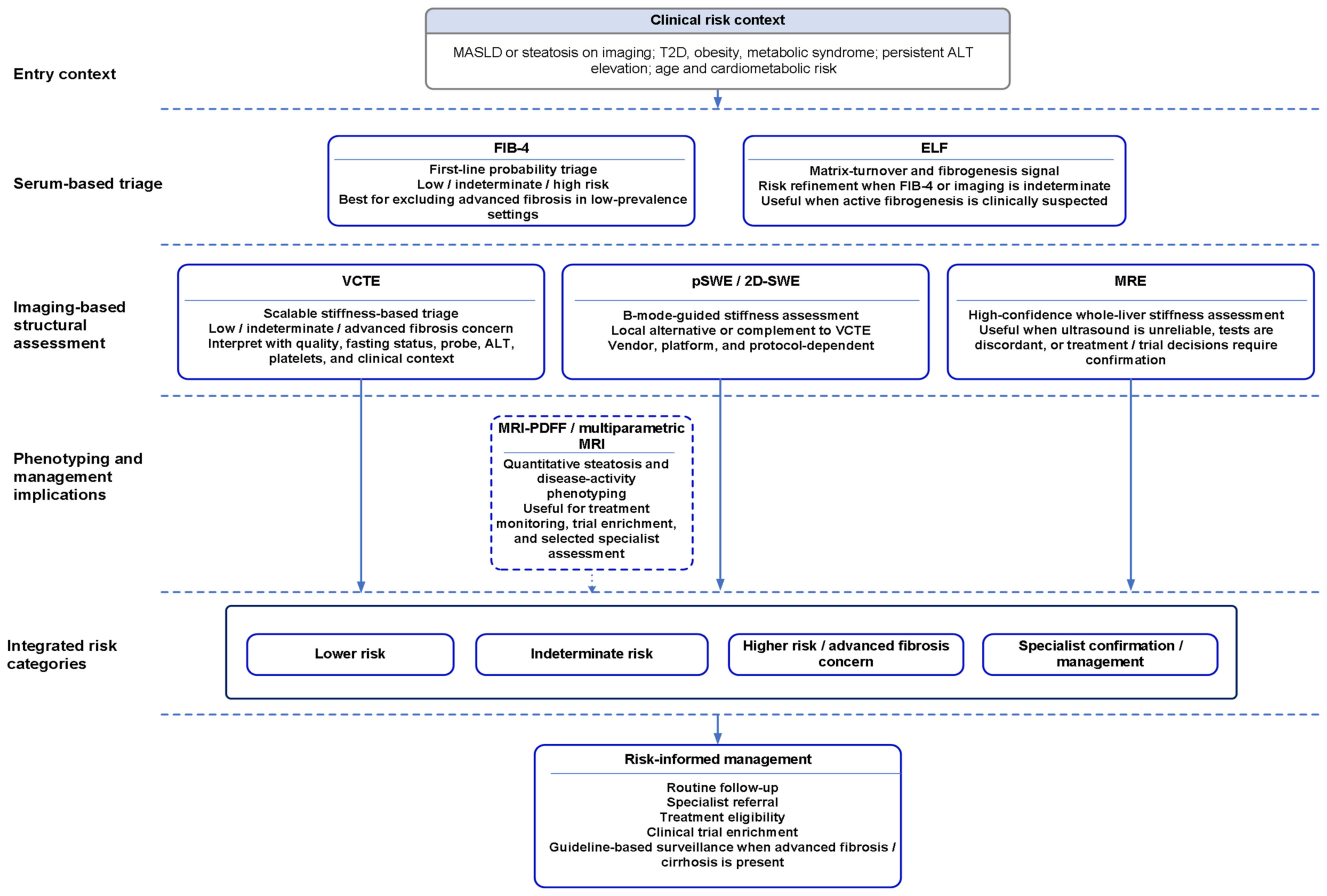
Integrated non-invasive risk-stratification framework in MASLD. Serum-based tests and imaging methods provide different layers of information rather than interchangeable results. FIB-4 is used mainly for initial probability triage, while ELF reflects matrix turnover and may help refine risk when first-line assessment is indeterminate or discordant. Stiffness-based imaging with VCTE, pSWE, 2D-SWE, or MRE then adds structural information, with MRE reserved mainly for unreliable ultrasound examinations, discordant tests, or decisions requiring greater diagnostic confidence. MRI-PDFF and multiparametric MRI may further support steatosis quantification, disease-activity phenotyping, treatment monitoring, and trial enrichment in selected settings. These inputs can be integrated into practical risk categories that guide follow-up, referral, treatment eligibility assessment, trial consideration, and guideline-based surveillance when advanced fibrosis or cirrhosis is present. The framework is intended to show complementary information sources, not a requirement that all tests be performed in every patient. Test selection should depend on availability, baseline risk, examination reliability, discordance among results, and whether the result is likely to change management. ALT, alanine aminotransferase; ELF, enhanced liver fibrosis test; FIB-4, fibrosis-4 index; MASLD, metabolic dysfunction-associated steatotic liver disease; MRE, magnetic resonance elastography; MRI, magnetic resonance imaging; MRI-PDFF, magnetic resonance imaging proton density fat fraction; pSWE, point shear wave elastography; 2D-SWE, two-dimensional shear wave elastography; T2D, type 2 diabetes; VCTE, vibration-controlled transient elastography.

Figure 2 summarizes an integrated framework combining serum-based tests, elastography, and MRI for risk stratification in MASLD. In practice, a useful combined strategy uses serum markers to estimate pre-test probability, imaging to refine structural risk, and selected composite tools when the clinical question involves active fibrogenesis, at-risk steatohepatitis, or high-stakes treatment decisions (1, 6, 210–212). Integrated assessment is most useful when serum markers, elastography, and MRI contribute different biological information rather than repeating the same signal.

## 12 Clinical pathways and decision-making

Imaging-based assessment in MASLD is most useful when it is placed within a stepwise pathway rather than used as an isolated test (180, 213). In primary care, diabetes, obesity, and metabolic clinics, the main task is to exclude advanced fibrosis in large numbers of at-risk patients. FIB-4 is well suited to this role because it is inexpensive, scalable, and easy to calculate from routine laboratory data (180, 213). A value below 1.3 generally supports a low short-term probability of advanced fibrosis, whereas indeterminate or elevated values should prompt second-line testing with VCTE or ELF, depending on local availability. In adults older than 65 years, a higher threshold, commonly above 2.0, may be used to reduce age-related false-positive classification (214–216).

At the second-line stage, the purpose is not simply to assign a fibrosis stage but to decide whether the patient can remain in routine follow-up or needs escalation (217–219). VCTE values below approximately 8 kPa generally support a low likelihood of advanced fibrosis when the examination is reliable and clinical suspicion is low. Values in the approximate 8–12 kPa range should be treated as indeterminate and interpreted together with platelet count, aminotransferases, metabolic risk burden, fasting status, and acquisition quality. VCTE values of approximately ≥12 kPa raise concern for advanced fibrosis or compensated advanced chronic liver disease and usually justify hepatology referral or confirmatory testing (63, 220). These thresholds are best used as practical ranges rather than universal constants.

A low FIB-4 value should not be interpreted as permanent reassurance in all patients. In individuals with low metabolic risk, repeat FIB-4 assessment at approximately 2–3-year intervals is generally reasonable. In contrast, patients with type 2 diabetes, obesity, multiple metabolic risk factors, persistent liver enzyme elevation, or imaging evidence of steatosis should undergo earlier reassessment, commonly within 1–2 years. VCTE may also be considered despite a low FIB-4 when clinical suspicion remains high, particularly in patients with diabetes, multiple metabolic risk factors, or discordance between biochemical tests and imaging findings (214–216).

For patients with indeterminate FIB-4 but low and technically reliable VCTE, the immediate likelihood of advanced fibrosis is generally low. These patients may usually remain in primary or secondary care with metabolic risk reduction, repeat FIB-4 testing, and repeat elastography when risk factors progress or laboratory abnormalities persist. ELF testing may be added when available, especially if there is uncertainty about active fibrogenesis or if serum-based and imaging-based results are not fully concordant (214–219). By contrast, elevated VCTE values require contextual interpretation. When VCTE is high but platelet count is normal and ALT is elevated, clinicians should first review fasting status, measurement reliability, probe selection, and possible inflammatory or cholestatic confounders. Repeating VCTE after ALT improvement or clinical stabilization may prevent overclassification. If stiffness remains high, if platelet count declines, or if the patient has a high-risk metabolic profile, hepatology referral and confirmatory assessment with MRE should be considered (63, 108, 120, 127, 220).

Discordant first-line tests should be treated as a reason to revisit the clinical context, not as an automatic indication for MRE. A high FIB-4 with low and reliable VCTE is often seen in situations where the serum score is being driven by age, transient aminotransferase elevation, or thrombocytopenia from causes other than portal hypertension (214–216). The reverse pattern, low or indeterminate serum-marker risk with elevated VCTE, may reflect a stiffness confounder rather than true fibrosis progression, particularly in the setting of ALT flare, cholestasis, hepatic congestion, recent food intake, or suboptimal acquisition (12, 25, 52, 53). Patient phenotype also matters. Severe obesity, particularly class III obesity, narrow intercostal spaces, subcapsular ascites, and poor acoustic windows can reduce the reliability of ultrasound-based elastography (54–56). Before escalation, clinicians should review fasting status, probe selection, acquisition quality, platelet count, aminotransferase pattern, and whether the result is plausible in the broader clinical picture. MRE is most useful when the discordance remains clinically important after these factors have been assessed (108, 120, 127).

**Figure 3:**
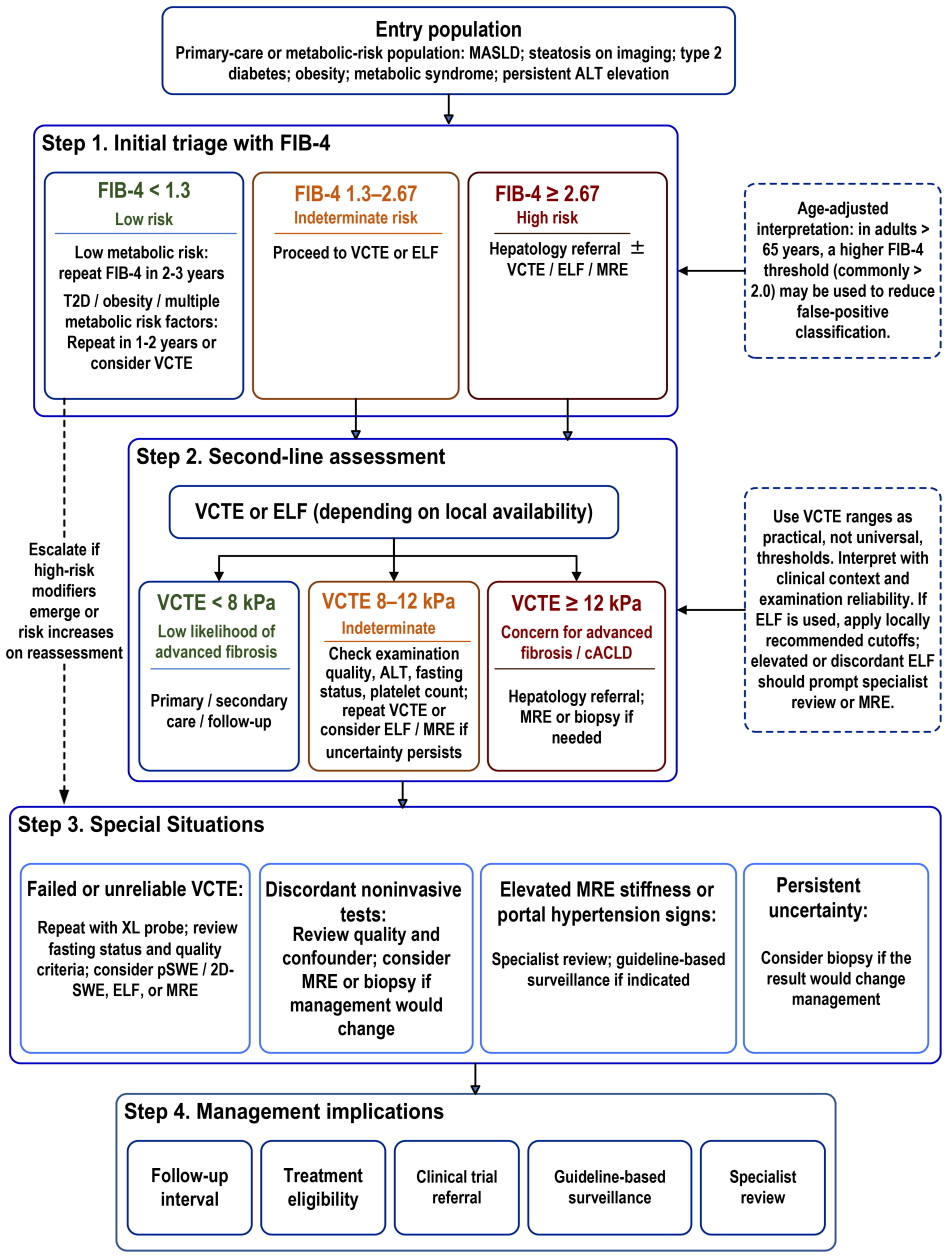
Suggested stepwise clinical pathway for fibrosis risk stratification and management in MASLD. The pathway begins with patients identified in primary-care or metabolic-risk settings, including those with MASLD, steatosis on imaging, type 2 diabetes, obesity, metabolic syndrome, or persistent ALT elevation. FIB-4 is used as the initial triage test, with low, indeterminate, and high-risk categories guiding follow-up or escalation. In patients with low FIB-4 but higher metabolic risk, such as type 2 diabetes, obesity, or multiple metabolic risk factors, earlier reassessment or VCTE may be considered. Indeterminate FIB-4 results generally prompt second-line assessment with VCTE or ELF, depending on local availability, whereas high FIB-4 values should prompt hepatology referral and additional non-invasive assessment when appropriate. VCTE ranges are shown as practical interpretive categories rather than universal thresholds and should be interpreted together with examination quality, fasting status, ALT, platelet count, clinical context, and reliability criteria. Failed or unreliable VCTE, discordant non-invasive tests, elevated MRE stiffness or signs of portal hypertension, and persistent diagnostic uncertainty should prompt repeat testing, alternative elastography, ELF, MRE, specialist review, or biopsy when the result would change management. Downstream management may include individualized follow-up intervals, treatment eligibility assessment, clinical trial referral, guideline-based surveillance when indicated, and specialist review. In adults older than 65 years, age-adjusted FIB-4 interpretation is preferred to reduce false-positive classification. ALT, alanine aminotransferase; cACLD, compensated advanced chronic liver disease; ELF, enhanced liver fibrosis test; FIB-4, fibrosis-4 index; MASLD, metabolic dysfunction-associated steatotic liver disease; MRE, magnetic resonance elastography; pSWE, point shear wave elastography; 2D-SWE, two-dimensional shear wave elastography; T2D, type 2 diabetes; VCTE, vibration-controlled transient elastography.

Failed or unreliable VCTE should first prompt reassessment of fasting status, probe choice, and acquisition quality. In severe obesity, repeat examination with an XL probe is usually the most practical next step. If reliable measurement still cannot be obtained, alternative strategies include pSWE or 2D-SWE performed by an experienced operator, ELF testing, or MRE when available (63, 108, 120, 127, 220). When VCTE and MRE are discordant, technical quality and confounders should be reviewed before one result is allowed to dominate. If both examinations are technically adequate, MRE generally provides the more reproducible whole-liver assessment and should usually carry greater weight in specialist decision-making (120, 124–127). However, liver biopsy remains appropriate when non-invasive tests remain inconclusive or discordant and the result would alter surveillance, treatment eligibility, trial enrollment, or the exclusion of alternative liver diseases (1, 6, 64). Figure 3 summarizes this stepwise pathway and its downstream management implications.

## 13 Limitations and unmet needs

Important limitations remain. Several issues continue to constrain imaging-based assessment in MASLD. First, no stiffness-based method is fully specific for fibrosis. Inflammation, congestion, and cholestasis may elevate stiffness and create false impressions of severity (12, 21, 25, 57). This limitation is not modality-specific but reflects a fundamental constraint of stiffness-based assessment. As a result, stiffness values should be interpreted within clinical context rather than as standalone indicators of fibrosis stage, particularly when results appear discordant with biochemical or clinical findings (221).

Second, obesity remains a major practical challenge. Ultrasound-based techniques are more susceptible to technical failure and reduced reliability in patients with increased body habitus, although probe adaptation and real-time guidance improve feasibility (54–56, 203). MRI is less affected but not immune to motion artifact, iron overload, and limited accessibility (6, 128–130, 203). These constraints reinforce the need for modality selection to be individualized rather than protocol-driven.

Third, standardization remains incomplete across imaging platforms. Cutoff values vary across techniques, vendors, and study populations (72, 73, 86–89). Even for MRE, proposed fibrosis thresholds may vary according to acquisition protocols, study populations, and analytical frameworks, and therefore should be interpreted as context-dependent reference ranges rather than universally transferable constants (120, 222, 223). This lack of harmonization limits cross-study comparability and complicates longitudinal interpretation in routine care.

Fourth, the evidence base for prognostic and treatment-monitoring applications is still evolving. Although liver stiffness is clearly associated with clinical outcomes, the optimal use of longitudinal change in individual patients remains uncertain (173, 180, 186, 187, 203). In particular, it is unclear how much change in stiffness reflects meaningful biological improvement vs. measurement variability or transient inflammatory effects (102, 173, 186, 187, 203). Future research should therefore prioritize linking imaging biomarkers to hard clinical endpoints and decision-relevant thresholds, rather than focusing solely on diagnostic performance.

## 14 Future directions

Future work in MASLD imaging will likely focus on multimodal integration, longitudinal monitoring, and linkage between imaging biomarkers and clinically meaningful outcomes. AI-assisted acquisition, automated segmentation, and quantitative MRI biomarkers may improve reproducibility and workflow efficiency, although external validation and standardization remain essential before routine implementation (224–230).

## 15 Conclusions

Imaging has become central to fibrosis assessment in MASLD because it makes non-invasive risk stratification clinically workable at scale (203, 213, 231). Ultrasound elastography remains the practical frontline tool, whereas MRE and quantitative MRI add precision when greater diagnostic confidence is needed (170, 230). Beyond baseline staging, imaging is increasingly incorporated into longitudinal risk assessment and treatment monitoring pathways (232–234). The most effective clinical strategy is usually sequential: serum-based exclusion first, elastography for refinement, and MRI reserved for unresolved or higher-stakes clinical questions (8, 203). This review emphasizes how imaging can be integrated into longitudinal, risk-based MASLD care through sequential testing, modality selection, and context-specific escalation pathways.
