# Supplementary material for: Imaging-based fibrosis assessment and risk stratification in MASLD
Source: Front Med (Lausanne). 2026 Jul 6;13:1880238. doi: 10.3389/fmed.2026.1880238 (PMC13382512; doi:10.3389/fmed.2026.1880238)
Supplement: Supplementary file 1 [file Table_1.docx]

Supplementary Material

# Supplementary Tables

**Supplementary Table S1. Practical comparison of imaging modalities for fibrosis assessment and treatment monitoring in MASLD**

| Modality / biomarker | Approximate diagnostic role | Common interpretive ranges or validated endpoints | Failure / reliability considerations | Main confounders | Cost / access | Reproducibility | Best validated endpoint | Role in monitoring | Best-fit clinical setting |
| --- | --- | --- | --- | --- | --- | --- | --- | --- | --- |
| Conventional B-mode ultrasound | Opportunistic detection of steatosis, cirrhotic morphology, and portal hypertension features; not reliable for early fibrosis staging | No validated fibrosis threshold; late findings include surface nodularity, coarse echotexture, lobar redistribution, splenomegaly, collateral vessels, or ascites | Operator dependent; limited by obesity, bowel gas, narrow acoustic windows, and subjective interpretation of morphology | Obesity, poor acoustic window, coexisting focal lesions, heterogeneous steatosis, and late-stage morphologic overlap | Low cost; widely available in primary and secondary care | Limited for quantitative fibrosis assessment; better for detecting gross morphologic change than early fibrosis | Detection of overt chronic liver disease or complications rather than fibrosis stage | Limited for fibrosis monitoring; useful for surveillance-related imaging when guideline indications are met and for detecting portal hypertension features | Primary and secondary care for initial abdominal assessment; not sufficient as a stand-alone fibrosis-staging test |
| VCTE | First-line imaging-based liver stiffness test for fibrosis risk stratification and referral triage | Commonly used MASLD ranges: <8 kPa suggests low likelihood of advanced fibrosis; 8–12 kPa is indeterminate; ≥12–15 kPa raises concern for advanced fibrosis or cACLD; ≥20–25 kPa may suggest cirrhosis/portal hypertension risk when supported by platelets and clinical context | Requires adequate valid measurements, appropriate probe, fasting state, median stiffness, and IQR/median assessment; failed or unreliable tests are more common in severe obesity or narrow intercostal spaces | ALT flare, active inflammation, cholestasis, hepatic congestion, recent food intake, obesity, probe mismatch, and poor acquisition quality | Relatively low cost; rapid, scalable, and widely deployable; best suited to population-level triage | Good when protocol, probe, fasting status, and quality criteria are consistent; weaker when platforms or acquisition conditions vary | Excluding or identifying advanced fibrosis/cirrhosis risk rather than distinguishing adjacent early fibrosis stages | Practical for serial follow-up, but small changes should be interpreted cautiously and confirmed when management would change | Primary and secondary care triage; referral decision support; tertiary care when used as part of a broader pathway |
| pSWE | Ultrasound-integrated focal stiffness assessment with B-mode guidance; alternative or complement to VCTE where local expertise exists | Platform-specific thresholds; may be reported in kPa or m/s; cutoffs are not directly interchangeable across vendors or disease settings | ROI placement, sampling depth, breath-hold, avoidance of vessels or focal lesions, and local quality criteria are essential | ROI depth, subcapsular artifacts, rib shadowing, probe pressure, obesity, inflammation, cholestasis, congestion, and operator technique | Available on some conventional ultrasound systems; access depends on equipment and trained operators | Moderate to good within the same platform and protocol; lower transferability across vendors than VCTE | Detection of significant or advanced fibrosis in locally validated settings | Useful for follow-up only when the same platform, protocol, and operator standards are maintained | Secondary care, ultrasound-based liver clinics, and centers without a separate FibroScan pathway |
| 2D-SWE | Ultrasound-integrated stiffness mapping with B-mode guidance; broader sampling than pSWE within a conventional ultrasound examination | Platform-specific thresholds; values may be reported in kPa or m/s; conversion between units assumes simplified tissue properties and may not be interchangeable | Requires stable breath-hold, appropriate elasticity map, careful ROI placement, and avoidance of artifacts; quality metrics vary by vendor | Depth, ROI placement, subcapsular artifacts, rib shadowing, reverberation, probe pressure, obesity, inflammation, cholestasis, and congestion | Available in advanced ultrasound platforms; operator and vendor dependence remain important | Good within standardized local protocols; cross-platform reproducibility is limited | Detection of advanced fibrosis when acquisition is standardized and locally validated | Potentially useful for serial monitoring, but only with strict platform and protocol consistency | Secondary or tertiary ultrasound-based liver assessment; locally validated alternative to VCTE |
| MRE | Higher-tier confirmatory imaging test with high diagnostic confidence and whole-liver stiffness assessment | Individual-patient meta-analysis cutoffs include approximately 3.14 kPa for ≥F2, 3.53 kPa for ≥F3, and 4.45 kPa for F4; higher values carry prognostic information | Requires dedicated hardware, software, technical expertise, and adequate image quality; limited by iron overload, motion artifact, severe claustrophobia, device contraindications, and local availability | Inflammatory activity, technical protocol differences, iron overload, motion, and histologic sampling limitations near lower fibrosis thresholds | High cost; limited access; usually available in tertiary or specialist centers rather than broad screening settings | High when acquisition and post-processing are standardized; better whole-liver reproducibility than ultrasound-based tests | Advanced fibrosis/cirrhosis staging, prognostic risk stratification, and trial-grade stiffness quantification | Preferred when serial quantitative stiffness is important, especially in trials, severe obesity, discordant tests, or unreliable ultrasound | Tertiary care, specialist phenotyping, clinical trials, high-stakes treatment eligibility, biopsy decisions, and discordant first-line testing |
| MRI-PDFF | Quantitative biomarker of hepatic steatosis; not a direct fibrosis marker | PDFF ≥5% generally supports steatosis; relative reduction of ≥30% is commonly used in trials as a steatosis-response or biological-response signal | Requires standardized MRI acquisition and analysis; interpretation depends on scanner protocol and fat quantification method | Iron, motion artifact, technical protocol variation, and changes in steatosis that may not parallel inflammation or fibrosis | Higher cost and lower access than ultrasound; often available in specialist or research settings | High for hepatic fat quantification when protocols are standardized | Steatosis quantification and treatment-response enrichment rather than fibrosis regression | Useful for monitoring liver fat response after lifestyle, bariatric, incretin-based, THR-β agonist, or other MASH-directed interventions | Clinical trials, proof-of-concept studies, specialist care, and selected treatment-monitoring scenarios |
| Multiparametric MRI / cT1 | Combined tissue characterization of steatosis, stiffness, and fibroinflammatory activity; useful for phenotyping complex disease | PDFF quantifies fat; MRE quantifies stiffness; cT1 and related biomarkers may reflect fibroinflammatory activity, but thresholds are less standardized | Requires harmonized acquisition and post-processing; cT1 and related markers are more vendor- and pipeline-sensitive than PDFF or MRE | Scanner differences, post-processing pipeline, inflammation, iron, edema, motion artifact, and differences in biological processes captured by each biomarker | High cost; limited availability; mostly specialist or research use | Variable by biomarker; PDFF and MRE are more reproducible than less standardized fibroinflammatory markers | Baseline phenotyping, trial enrichment, and distinguishing steatosis response from broader disease activity | Useful when treatment response may involve steatosis, activity, and stiffness changing on different timelines | Tertiary care, research, clinical trials, and complex cases with discordant biomarkers |
| CT / quantitative CT | Adjunctive or opportunistic assessment of advanced chronic liver disease features; not a primary MASLD fibrosis-staging test | Conventional CT detects late morphologic signs; quantitative CT, extracellular volume, texture, and liver surface nodularity metrics remain investigational | Radiation limits repeated use; contrast timing, acquisition protocol, reconstruction method, and segmentation affect quantitative measures | Contrast phase, scanner protocol, steatosis, congestion, focal lesions, body habitus, and comorbid liver disease | Widely available when CT is clinically indicated; not cost-effective or appropriate as a dedicated fibrosis-monitoring tool | Variable; depends on protocol standardization and analytic method | Detection of advanced morphologic disease or opportunistic risk signals rather than early fibrosis staging | Limited for serial fibrosis monitoring because of radiation and protocol variability | Opportunistic or adjunctive use in patients undergoing CT for other indications; not a first-line MASLD fibrosis pathway tool |
| Radiomics / AI tools | Emerging adjunct for automated fibrosis classification or multimodal risk prediction; not a replacement for established NIT pathways | No accepted clinical thresholds; performance commonly reported by AUROC, sensitivity, specificity, calibration, and decision-curve analysis | Limited by retrospective design, curated datasets, segmentation variability, vendor/scanner heterogeneity, poor explainability, and limited prospective validation | Acquisition protocol, scanner/vendor, segmentation method, population shift, disease prevalence, class imbalance, and spectrum bias | Not standard care; implementation requires software, workflow integration, validation, governance, and regulatory evaluation | Uncertain across sites unless externally validated and calibrated; reproducibility depends on model transparency and data quality | Model discrimination and risk prediction remain common endpoints; outcome improvement and decision impact are less often proven | Research tool; potential future role in integrating imaging, laboratory, and EHR data for risk-stratified pathways | Research settings, externally validated decision-support tools, and future integrated care pathways; not routine primary-care triage |

Note: AI, artificial intelligence; ALT, alanine aminotransferase; AUROC, area under the receiver operating characteristic curve; cACLD, compensated advanced chronic liver disease; cT1, corrected T1; EHR, electronic health record; MASLD, metabolic dysfunction-associated steatotic liver disease; MASH, metabolic dysfunction-associated steatohepatitis; MRE, magnetic resonance elastography; MRI-PDFF, magnetic resonance imaging proton density fat fraction; NIT, noninvasive test; pSWE, point shear wave elastography; ROI, region of interest; 2D-SWE, two-dimensional shear wave elastography; THR-β, thyroid hormone receptor-beta; VCTE, vibration-controlled transient elastography.

**
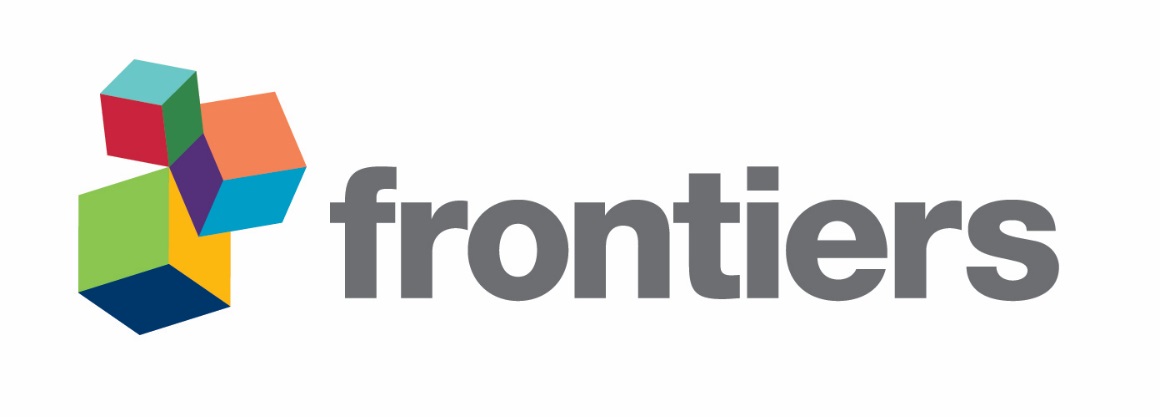
**
